# A case report of aggressive mixed epithelial and stromal tumor of the kidney with malignant transformation

**DOI:** 10.1016/j.eucr.2024.102689

**Published:** 2024-02-22

**Authors:** Anahita Ansari Djafari, Hossein Rahnama, Babak Javanmard, Seyyed Ali Hojjati, Sareh Salarinejad

**Affiliations:** a Laser Application in Medical Sciences Research Center, Shohada-e-Tajrish Hospital, Shahid Beheshti University of Medical Sciences, Tehran, Iran; b Department of Urology, Shohada-e-Tajrish Hospital, Shahid Beheshti University of Medical Sciences, Tehran, Iran; c Department of Pathology, Shohada-e-Tajrish Hospital, Faculty of Medicine, Shahid Beheshti University of Medical Science, Tehran, Iran

**Keywords:** Kidney, Mixed epithelial and stromal tumor, Renal mass, Sarcomatoid component

## Abstract

Mixed epithelial and stromal tumor of the kidney (MESTK) is a rare benign kidney tumor. In rare cases, malignant transformation, such as sarcomatoid features indicates poor clinical outcomes.

In this study, we will describe a 45 years old man with a diagnosis of MESTK with malignant transformation of the sarcomatoid component, after right radical nephrectomy. The patient underwent chemotherapy with adriamycin, ifosfamide, and granulocyte-colony stimulating factor (G-CSF).

The radiological characteristics of MESTK can pose diagnostic challenges due to its non-unique radiological appearance. The presence of sarcomatoid transformation is a hallmark feature of malignant MESTK which can be very aggressive.

## Introduction

Mixed epithelial and stromal tumor of the kidney (MESTK) is a rare benign tumor that predominantly affects women. In fact, it is approximately 7 times more common in women than in men and is often diagnosed in perimenopausal women or following long-term estrogen therapy. The age range of patients at diagnosis is quite broad, ranging from 19 to 78 years, with an average age of around 52 years. MESTK is typically discovered incidentally, and its most common symptoms include flank pain and hematuria. This condition has been referred to by various names in medical literature, such as adult-type mesoblastic nephroma, cystic hamartoma of the pelvis, adult-type cystic nephroma, leiomyomatous hamartoma, and solid and cystic biphasic tumor of the kidney.^1^

Computed Tomography (CT) scan and Magnetic Resonance Imaging (MRI) can help to diagnose these masses, but the definitive diagnosis will still be based on pathology findings. Cystic with heterogeneous and delayed contrast enhancement in the septations and solid components is how these lesions are described in CT scan. The majority of these lesions fall into Bosniak category III or IV.^2^

Mixed epithelial and stromal tumors (MESTK) of the kidney are rare neoplasms that exhibit a biphasic growth pattern consisting of both epithelial and stromal components. While most MESTKs are benign, in rare cases malignant transformation, such as sarcomatoid features, has been reported, which indicates poor clinical outcomes. In this study we will describe a 45 years old man with a diagnosis of MESTK.

## Case report

A 45-year-old man referred to the urology clinic due to right flank pain. During the examination, a large mass was palpable on the patient's right flank. He did not mention any history of gross hematuria or weight loss and had no history of underlying malignancy or hormone therapy. The patient had normal male secondary sex characteristics. In terms of laboratory tests, urine analysis and blood routine tests were normal with a Hemoglobin level of 13.6 g/L and Creatinine level of 1.4 mg/dl. Ultrasound showed a hypoechoic lobulated mass measuring 120 × 80 milimeter (mm) in the upper pole of the right kidney.

**Fig. 1:**
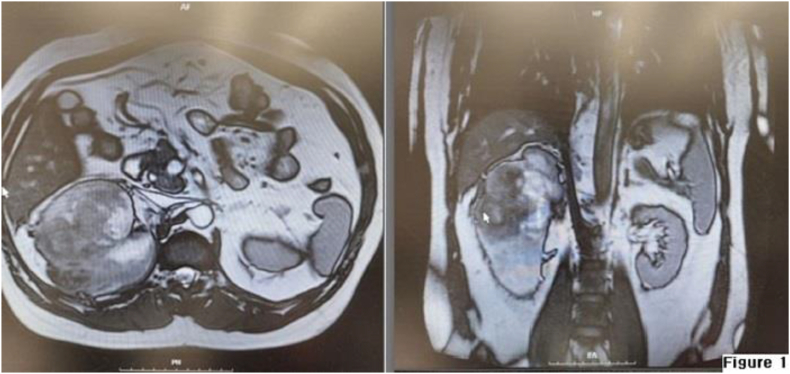
Abdominopelvic MRI showed 101 × 92 mm mass in upper-middle pole of right kidney.

During the MRI, a mass with irregular and unclear borders was detected in the upper-middle pole of the right kidney, measuring approximately 101 × 92 mm. The mass displayed heterogeneous signal intensity both on T1-weighted and T2-weighted images. The mass had extracapsular extension and adhesion to the liver capsule. There was no evidence of lymphadenopathy or involvement of the renal vein or the hepatic flexure of the colon. (Fig. 1).

Chest CT scan showed no evidence of metastasis. The patient underwent right radical nephrectomy. According to the histopathology findings, the spindle cells surrounding the epithelial component displayed features consistent with a MESTK. The sarcomatoid component also showed malignant transformation. The tumor invaded the perinephric fat and diaphragm, however there has been no evidence of lymphovascular invasion. The pathology of the soft tissue and diaphragm wall mass suggests that the tumor has also spread into skeletal muscle and soft tissue. The pathological stage was PT4bN0M1.

**Fig. 2:**
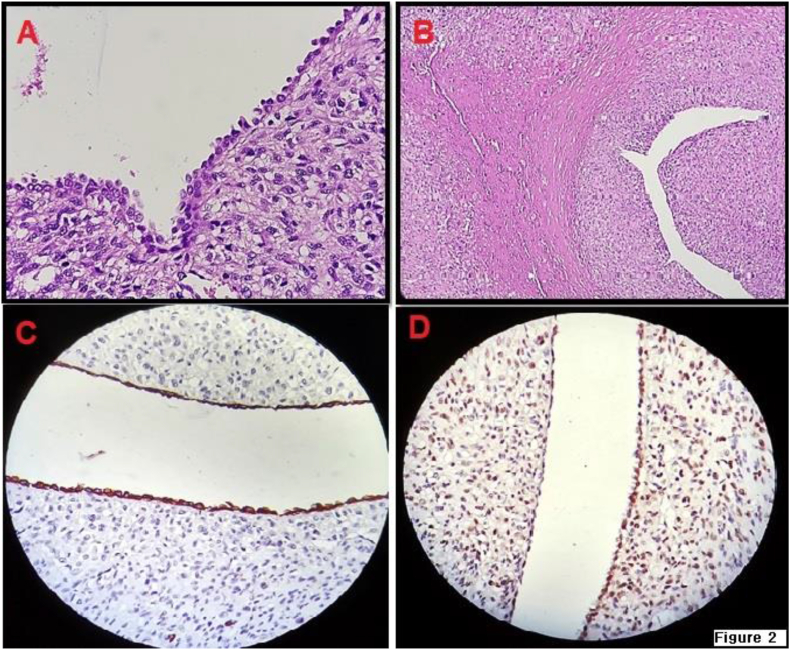
HE Staining, epithelial and stromal components, with spindle cells condensed around the epithelial component. Epithelial cysts are lined by flat, cuboidal, columnar, or hobnail cells. (A) Sarcomatous transformation (cytologic atypia, necrosis, and mitosis) (B) Immunohistochemistry (IHC). Cytokeratin AE1/AE3:Positive in glandular epithelial cells (C). Progestrone Receptor (PR): Positive in glandular epithelial cells and stromal elements (D).

Based on the immunohistochemistry (IHC) examination, the glandular epithelial cells were found to be positive for Cytokeratin AE1/AE3, while both the glandular epithelial cells and stromal elements showed positivity for Progesterone Receptor (PR). (Fig. 2).

**Fig. 3:**
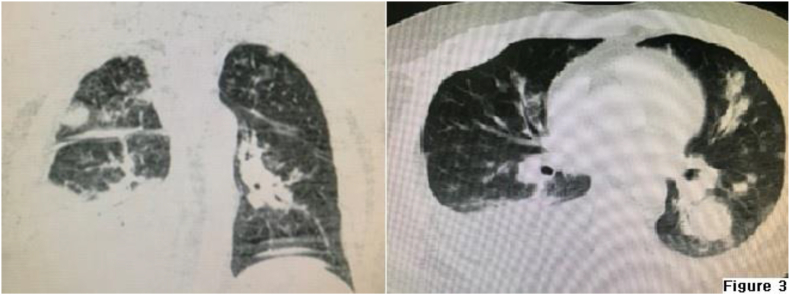
Lung metastasis in Chest CT scan, one month after the surgery.

The patient presented with shortness of breath one month after surgery and was found to have a large pleural effusion on the right side. No evidence of malignancy was found in cytopathology of pleural fluid. However, the radiology report indicated the presence of metastasis (Fig. 3).

Whole-body bone scan was performed and multiple bone metastases reported. The patient underwent chemotherapy regimen consisting of adriamycin, ifosfamide, and granulocyte-colony stimulating factor (G-CSF), but he did not respond properly to chemotherapy and died within 6 months due to the extent of metastases and the progressive course of the disease.

## Discussion

The low incidence of malignancy in MESTK makes it challenging to establish the true clinical behavior and prognosis of this rare tumor type.^3^ The radiological characteristics of MESTK can pose diagnostic challenges due to its non-unique radiological appearance. The tumor may present as a solid or cystic renal mass, and the cystic component is often dominant.^4^ In the case of our patient, the MRI findings were suggestive of malignancy, but they did not provide a definitive diagnosis of MESTK. Surgical excision and histopathological examination may be necessary to confirm the diagnosis.

Percutaneous biopsy can be considered, but it may be challenging due to the limitations of the sample size. The presence of sarcomatoid transformation is a hallmark feature of malignant MESTK.^3^ The diagnosis of malignant MESTK requires the presence of distinct epithelial elements with an associated stromal cuff, which is characteristic of MESTK. The presence of sarcomatoid transformation is a hallmark feature of malignant MESTK.^3^^,^^5^

In the immunohistochemistry examination, the presence of tumor markers such as smooth muscle actin (SMA), Desmin, Caldesmin, CD10 and Esterogen Receptor (ER) will favor the diagnosis of MESTK. In patients with positive ER and PR, history of hormone therapy is more common.^1^^,^^3^

Some limited studies suggest that MESTK with evidence of malignant transformation is sensitive to palliative chemotherapy with doxorubicin and ifosfamide. Recurrence-free survival of some patients has been reported up to 36 months.^3^^,^^5^ In the case of our patient, due to multiple metastases, chemotherapy with adriamycin and ifosfamaide was started.

## Conclusion

The coexistence of sarcomatoid components in patients with MESTK underscores the significance of thorough pathological examination in individuals with cystic renal lesions, particularly those with MESTK. The presence of sarcomatoid components in MESTK patients portends a poor prognosis due to the aggressive nature of these components and can result in metastasis and unfavorable survival outcomes. The sensitivity of this type of MESTK to palliative chemotherapy with doxorubicin and ifosfamide has been reported, but additional research is required to establish optimal treatment strategies.

## Ethics

Patient informed consent was obtained to publish his information. The patient's private information remained confidential with the researchers.

## Financial support and sponsership

None.

## CRediT authorship contribution statement

**Anahita Ansari Djafari:** Software, Methodology, Conceptualization. **Hossein Rahnama:** Writing – original draft, Visualization, Investigation. **Babak Javanmard:** Validation, Supervision, Software. **Seyyed Ali Hojjati:** Data curation. **Sareh Salarinejad:** Writing – review & editing.

## Declaration of competing interest

The authors declare no conflicts of interest in this work.
